# Leisure Behavior of Young Immigrants in Andalusia (Spain): The Process of Acculturation through Physical Activities and Sport

**DOI:** 10.3390/ijerph19010580

**Published:** 2022-01-05

**Authors:** Juan Carlos Checa, Ángeles Arjona, Montserrat Monserrat, Darío Salguero

**Affiliations:** 1 Department of Geography, History and Humanities, Faculty of Humanities, University of Almería, 04120 Almería, Spain; arjona@ual.es; 2 Social and Cultural Anthropology Laboratory, Faculty of Humanities, University of Almería, 04120 Almería, Spain; mmh548@inlumine.ual.es; 3 Department of Education, Faculty of Education, University of Almería, 04120 Almería, Spain; dariosalguero@ual.es

**Keywords:** immigrants, sport, physical activity, leisure time, acculturation, integration, Spain

## Abstract

The purpose of this paper is to understand the role that physical activity and sport plays during leisure time (LTPAS) in the social integration of young immigrants (Africans, Latin-Americans, and Eastern Europeans) in Andalusia, Spain. Method: With this aim, Physical Activity and Sport Acculturation Index (PASAI) data were collected through a survey of the immigrant population aged 15–20. The final sample consists of 440 surveys. The average age was 17.6 (SD = 2.9). 48.4% of them were men, 72% were single, and 72.8% had secondary-level studies. In terms of generation, the second-generation population represented 25.8% of the total, the 1.5 generation 43.5%, and the first generation 30.7%. The questionnaire was voluntarily answered by immigrant students in classrooms and was completed in the second stage via random surveys of residential areas to cover the quota of age and origin. A regression analysis was applied in two phases, generating two models. The first included independent socio-demographic variables; the second included structural variables. Results: First, the results show that immigrants have a low participation rate in physical activity and sport during their leisure time. Second, generation and origin are the main variables that predict variation in physical and sport participation.

## 1. Introduction

Spain has ceased to be a country of emigration and has become a host society [1]. According to the Spanish National Statistics Institute (INE) [2], in the 1990s there were 542,314 foreign people living in Spain, in the year 2000 there were 1,370,657, and today there are 5,373,917.

The importance of this phase of migration is that, with the increasing flows of people, there is a greater deal of cultural diversity. Each group of immigrants brings a cultural heritage defined by language, religion, work, family, leisure experiences, etc., that must be adapted to the new environment. That is the reason why the major problems faced by immigration host countries are how to regulate the flows of immigrants and, above all, how to manage the integration of the new population [3,4,5]. Their social integration depends on how “contexts of reception” combine government policies, societal reception, and co-ethnic community [6,7]. 

Social integration is a multidimensional process that involves many variables: employment, housing, gender, health, and leisure [4,8,9]. More recently, physical activity and sport (PAS) as part of leisure time (LTPAS) has been considered by a significant number of researchers to be a fundamental element in the social integration of immigrants, inter-group communication [10,11,12], and in challenging disparities and xenophobia [11,12,13]. Moreover, in recent years, PAS has become part of the political measures adopted to improve the social integration of immigrants [14,15].

However, some critics show that PAS is not an automatic integration tool, since it must be accompanied by well-planned educational actions. Thus, PAS can actually obstruct social integration if it is not properly applied, bringing about violence, lack of communication, segregation, exclusion, the rejection of others, and ethnic withdrawal [11,15]. In those cases, immigrants will tend to maintain their original ethnic identity, internal solidarity, and ethnic boundaries.

Spain—unlike other countries with a longer experience of immigration, such as France, Germany or the United Kingdom—does not have players of second or third generation in its national football team. Therefore, sporting activities do not yet play a role in group cohesion and national identity in Spain. 

For that reason, the expectations generated by LTPAS as a mechanism for the social integration of immigrants has given rise in Spain to a number of investigations [11,12,14,16,17] of tournaments and sports competitions organized by immigrant associations, social educators, foundations, universities and/or public administrations [11,18,19,20], to evaluate their effects on the social integration process. In almost every case, the evaluation is based on the degree or number of immigrant participants as well as the design of the activity: whether mixed or homogeneous groups. The outcomes, in general, show that LTPAS involves a risk of exclusion, which can be multiplied if it is not developed from an intercultural orientation, since in many cases the result has been ethnic withdrawal: LTPAS turns into “us” (immigrants) versus “them” (native). Therefore, it can develop into rivalry rather than cooperative relationships.

Different reasons have led us to use, for the empirical part of this study, the concept of acculturation rather than integration. First, social integration is a concept which, as noted above, covers many aspects of the social and economic life of immigrants. Second, LTPAS research is primarily focused on the activity of immigrants, without examining in detail the role of the reception context, as proposed by the ecological model [21]. Third, most of the variables have a strong cultural and relational nature (with the locals and compatriots). Hence, acculturation, understood as the phenomena and changes resulting from direct and continuous contact between population groups, is a more appropriate concept to explain the participation of immigrants in LTPAS. Moreover, because the two concepts do not necessarily imply the same situation (that is, immigrants may be acculturated in LTPAS but not integrated), it must be recognized that acculturation can be a step towards social integration.

The objective of this research is to measure the acculturation of young immigrants settled in Andalusia, Spain via LTPAS. For this purpose, it applies the Physical Activity and Sport Acculturation Index (PASAI) [22,23], for which the main areas of research into the process of social integration are as follows: norms, cultural references, mixed relations, and transnationality [24,25,26,27]. Variables that refer to the LTPAS have also been included in each area.

In short, with this study we want to consolidate a new line of research in Spain based on the use of the PASAI as a measuring instrument and origin and generation as the two main variables, and to find out how these elements are configured in differential sport acculturation. (For an exhaustive review of research about ethnicity and leisure, see Stodoslka [28,29].)

### Theoretical Framework

The theoretical and conceptual framework guiding this research is based, in part, on the different theoretical models that explain the acculturation of immigrants. It also analyzes the role of variables such as sex, religion, and socioeconomic status in the employment of leisure time.

For much of the last century, assimilation theory dominated the literature on immigrants [27]. This theory assumes that there is a natural process by which different ethnic groups end up sharing a common culture and acquire a similar structure of opportunity in society [7]. Gordon [21] indicated that this situation occurs in several phases. First, immigrants begin their adaptation through acculturation. Minority groups acquire the patterns of the majority, especially with regard to cultural references such as language, religion, food, sport habits, and use of leisure time, among others (identifiable assimilation). 

Second, immigrants experience a structural assimilation. Indicators of this include intermarriage and the extent to which the social characteristics of individuals mix within neighborhoods, work situations, and leisure situations.

The last phase is called by Gordon [21] civic assimilation: immigrants share values and norms with the native population, e.g., emancipation of women, being able to choose their occupation, type of marriage relationship, and even a low level of religious practice. In this way, friction elements disappear, and a common identity is created. 

In short, the assimilation perspective predicts that over a number of generations the descendants of immigrants will be more akin to the host culture than their parents or grandparents.

More specifically, this perspective assumes that the immigrant population will be incorporated into the PAS practices of the native population by participating in their sports competitions and by using existing facilities to build a shared collective identity [17,22,23,28]. 

Within this perspective, there are two ways of managing cultural pluralism. It should be noted, first, that multiculturalism refers to societies that incorporate a heterogeneous collection of ethnic and racial groups in which immigrants actively shape their own lives [27,30]. However, when the state leaves (consciously or not) the management of diversity to the individuals, essentialism can appear and naturalize cultural differences. This is the basis for racist cultural fundamentalism and ideas of inferiority and superiority among different cultures. 

Second, the interculturalist trend recognizes the equality of groups and cultures in terms of symmetry [27,30]. Interculturalism puts more emphasis on mutual learning, cooperation, and exchange, including the peaceful regulation of inter-ethnic unrest. Thus, in the field, LTPAS proposes exchange and joint participation between the native and foreign populations [31,32,33]. Despite differences, Portes [34] points out that previous models are not able to explain why there are different paths of accommodation between different immigrant groups. He argues that previous explanations were too homogeneous and linear because they assumed that all population groups experienced the same path. However, reality has shown that, depending on the origin or the generation, as well as other variables, the integration of immigrants differs. Thus, Portes [34] proposes an alternative model called segmented assimilation theory. This maintains that there are three possible paths of adaptation among first-generation immigrants and their children. The first involves a process of acculturation and integration into the country’s middle class, which coincides with the classical concept of assimilation. The second takes the opposite direction; that is, a situation of permanent poverty and assimilation with the underclass. The third states that, despite possible economic progress, immigrants choose to deliberately preserve their own values of origin through their communities’ social networks and solidarity within their groups (selective acculturation). Consequently, this theory consists of a process involving multiple factors (family, individual, and contextual) that come about, not separately, as classic assimilationist theorists conceived it, but as a result of the interaction of all these levels [8].

Since the segmented assimilation theory, a new element of analysis has been introduced in the study of the social integration of immigrants: transnationality. It has appeared in response to research focused exclusively on the analyses of migration in the host society. Glick Schiller, Basch, and Szatón-Blanc [35] show that immigrants do not break ties with their countries of origin; on the contrary, fluid economic, social, and political relations remain at the same time that immigrants are integrated into the host society. Guarnizo [25,26] even argues that these ties do not occur only between two territorial units (bilateral or translocal relations between origin and destination), but that immigrants also have connections with other immigrants located in multiple destinations. 

One of the possible consequences of transnational lifestyles in the cultural field is prevention against assimilation. Moreover, immigrants can generate a process of re-identification with their places of origin [36,37]. As a result of transnationalism, immigrants not only do not abandon their original identity, but can strengthen it regardless of the path followed in other aspects of social integration. In other words, immigrants can be economically successful and accept as their own standards those of the host society, but, at the same time, the continuous contact with home can cause their identity to be more akin with their culture of origin. Thus, in LTPAS, the immigrant, including the second and subsequent generations, can practice physical activities appropriate to the country of arrival, while being part of teams representing the country of origin (community sport).

Thus, the LTPAS participates in the consolidation of their social identity, which is understood as “the knowledge possessed by an individual belonging to certain social groups together with the emotional significance and value of that membership” [38]. Therefore, depending on the identity that an individual acquires, he or she will show a type of behavior regarding alterity.

Finally, as we will show, there are several variables that correlate significantly with LTPAS. International research has paid special attention to religion [39,40,41,42,43] and sex [29,43,44,45].

## 2. Materials and Methods

### 2.1. The Participants

The research was conducted in the eight provinces of Andalusia in 2019. The target immigrant population of 52,383 was delimited by three criteria: first, by the towns and cities of Andalusia with a high density of immigrants (12%) (population in the census of 2020); second, by population aged 15–20, which represents the highest concentration of young immigrants in Spain [2,46,47,48]. However, youngsters who, even within the age limits (aged 15–20), did not fulfill the other requirements—for instance, minors without tutelage—were excluded because it was impossible to contact them: they are not in school, nor in minors’ centers. In fact, it is an “almost invisible” sample. The third criterion of delimitation was by population that comes from Latin America, Eastern Europe, and Africa. Africans were divided for this study into two groups: Maghrebis and Sub-Saharans. This distinction is made according to two criteria. The first of these was race: North Africans are mostly white; Sub-Saharan Africans mostly black. The second of these was religion: North Africans are mainly Muslims, while many Sub-Saharan Africans are Christians, animists, etc., with some Muslims among them, too.

The final sample consisted of 440 surveys. Key features of the sample show that the average age was 17.6 (SD = 2.9), 48.4% were men, 72% were single, and 72.8% had secondary level studies. In terms of generation, we have followed the classification proposed by Portes and Rumbaut [7], who define a “pure” second generation as made of the children of immigrants born in Andalusia (25,8%). The children who arrived in Andalusia aged under 16 (We have chosen the age of 16, and not 14, as Portes and Rumbaut [7] suggest, because the Spanish educational system establishes that age as mandatory for schooling. Once the student is older, he or she can abandon their studies and be incorporated into the labor market) are defined as 1.5 generation (43.5%). Finally (When one of the parents is foreign and the other is Spanish, the child is considered 2.5 generation, but in this research there were no cases of these), those who arrived when they were older than 16 years of age are considered first generation (30.7%). In general, African origin was represented by Moroccans and Senegalese; Latin American by Ecuadorians; Eastern European by Romanians (Table A1). As for social class, the average income of immigrants is 857 € (SD = 345) and 52.5% belong to a lower social class. 

### 2.2. Testing Procedure

The surveys were administered in person, with a proportional stratification of participants regarding age and origin. A multistage sample was used. First, surveys were conducted in schools with immigrants representing at least 12% of enrolled students. In each high school, the questionnaire was administered to students of secondary education (15–18 years); the classrooms were randomly selected among the three itineraries established in Spanish formal education. The questionnaire was voluntarily answered by students of immigrant background in these classrooms.

Second, since the Spanish education system has mandatory schooling to the age of 16, and the age range of our study was from 15 to 20, sampling was completed, in the second stage, via random interviews in residential areas to cover the quota of age and origin.

Finally, the confidence level of the survey was 95.5% with an estimation error of ±4.65 and a maximum variability (*p* = q = 0.5). The response rate was total.

### 2.3. Instrument and Variables

To measure the degree of sport acculturation in immigrants, an original index has been constructed known as the Physical Activity and Sport Acculturation Index (PASAI) [22,23]. This index examines key elements in the cultural values and social systems of immigrants in relation to LTPAS. Gordon [21] argues for the importance of the following factors in the process of assimilation: cultural references, mixed relationships, and norms. More recently, other authors have named transnationality as a relevant factor [25,26]. All the variables included in these dimensions make a highly significant contribution to the LTPAS.

Each dimension contributes two items to the index. These participate with the same weight, and were taken from other sports and immigration investigations and, in some cases, adapted to our field (Table A2).

From the response of each of the variables defining the dimensions is added the value 1 or 0. Value 1 was given to those alternative responses that, in theory, are likely to promote acculturation, inter-group communication, and sports participation, while value 0 was given to opposite situations. Hence, the index ranges between 0 and 8, in which a value of 0 indicates no acculturation, and a value of 8 indicates total sport acculturation. This means that there are no differences in PAS practice between immigrants and Spaniards. Below, each dimension is presented in a more specific way. 

As regards mixed relationships, we mean the friendship or comradeship established between immigrants and natives through physical activity [16,19,47,48,49]. The cultural references in PAS attempt to measure the frequency and the type of activity (universal vs. local). In the dimension of norms, it is the degree of participation and knowledge of PAS activities organized by different institutions that is being studied [16,20,47]. 

Finally, among the activities that define transnationality, we have chosen for this research, first of all, the frequency in which information is obtained about sports events in their country of origin; on the other hand, we also asked about the sense of national identity.

To check the reliability of the index, we performed two tests: the first through the technique of test and re-test, in which the Pearson r coefficient reached 0.71. For the second test we performed the Kuder Richardson 2.0, with a coefficient of 0.67. 

The independent variables introduced in the regression model, like those found in similar national and international research [12,16,19,20], correspond to both individual and structural levels. The first type included gender (1 = male), age, marital status (1 = single), origin (Sub-Saharan Africans were taken as base), family income (1 = low, less than €600 monthly), subjective social class (1 = high), house ownership (1 = owner), religion (Jews were taken as base), ideology (1 = left-wing ideology), number of languages spoken, knowledge and use of the language of the host society (1 = understand and speak properly), contact with origin (1 = yes), the idea of returning (1 = yes), current status (1 = enrolled at school), years of schooling, and generation (1 = first). 

In the structural level we included neighborhood composition (1 = mostly Spanish), father’s educational level, (1 = illiterate), identification (1= otherness), and number of household members. 

### 2.4. Data Analysis

Finally, we used the SPSS statistical program v.22 to analyze the data. The principle of normality used is Kolmogorov-Smirnov (*p* = 0.320). In the tables, the Chi-squared test (X^2^) between the dependent variable PASAI and the origin is 69.04; (gl = 21; *p* = 0.000); and with generation X^2^ = 72.03; (gl = 21; *p* = 0.000). 

To know the variability of the PASAI, a regression analysis was applied in two phases, generating two models. The first included independent socio-demographic variables; the second included structural variables. The coefficient R^2^ for the first model was 0.41, for the second 0.49. Both independent nominal and ordinal variables were converted into dummy variables.

### 2.5. Limitation Section

The results shown in the following section present a number of shortcomings. Firstly, PASAI has only been used in research carried out in Andalusia among its foreign population; it should also be used with foreign populations in other countries to check its validity. Secondly, to achieve a higher score in the index does not necessarily mean a higher degree of acculturation in the host society. Social integration constitutes a very complex and multifactorial process that goes beyond sport. The index only helps to build a new interpretation of a phenomenon.

## 3. Results

Table A3 shows that in mixed relationships the main reason why immigrant youth practice sports is health (64.1%), followed by pleasure (21.7%), and social relationships (16.1%). The practice is, in general, carried out individually (42.3%) or with people from the country of origin (31.6%).

In terms of cultural references, the option “1–2 days a week” was the most chosen answer regarding the frequency of sport practice (33.7%). As regards the type of LTPAS, 96.1% claimed to be universal type; that is, they perform an activity with universal rules that is practiced around the world, i.e., mass sport. However, 2.8% practice sports or traditional games that, except for exhibitions elsewhere, are only practiced in their countries of origin. 1.1% of the sporting activity consists mainly of exercises and games of Spanish origin. 

The variables about the norms show that the vast majority of foreign youngsters are not aware of public sports provision (90.3%) and only 9.7% participate in organized extracurricular school sports.

Finally, transnationality indicators show that 76.8% of young immigrants get PAS information from their country of origin or their parents’ country of origin, but only 10.2% get it daily or several times a week (22.1%). Also, 63% identify themselves as Spanish and foreign and only 36.3% as foreign, regardless of their birthplace.

The PASAI, as an inter-group cohesion element (Table A4), shows that the average is 3.3, a relatively low value given that the index ranges from 0 to 8. Even so, the first five scale values account for 90.6%. Africans, Sub-Saharans, and Maghrebis obtained the lowest score. However, Eastern Europeans and Latin Americans got different results; so, in the former case, the four highest values represent 13%; in the latter, 12.7%.

When the analysis took generation as its reference, we found that the more distance there is from the first generation, the greater the involvement of individuals in LTPAS (Table A5). Thus, if the first generation obtained a value of 16.3% in the 0 option, the figure drops to 3.1% in generation 1.5, and 0 in the second generation.

Finally, in the first regression model (Table A6), the main variable that describes changes in the index is the generation with a negative relationship. In other words, first generation immigrants in this study practice less LTPAS than 1.5 and second generation. The second variable is age, with a negative manifestation, since the older the subject is, the less LTPAS he or she does. The third is occupation, with a positive relationship, so that students are mostly active in LTPAS. Subsequently, a positive relationship was found for Latin Americans and Eastern Europeans, Christians and males; and a negative one for Muslim immigrants. Therefore, of the sample analyzed, the origin and certain associated cultural patterns, like religion, have a significant effect on the practice of PAS in young immigrants in Andalusia. To be precise, being Muslim and woman limited participation. Finally, the variables are linked to social class, income, subjective social class, and house ownership, which show that having lower incomes, belonging to a lower social class, and not being the house owner imply a lower level of acculturation in LTPAS.

The second model shows that immigrants residing in Spain (which constitute the majority) have a higher level of LTPAS practice. Moreover, to be recognized as “other” limits participation in LTPAS, as well as having an illiterate father and living in a house with many people.

Finally, it should be stressed in both models that variables such as North African origin, ideology, number of languages spoken, the idea of returning, atheists, and contact with the country of origin have only a marginal effect.

## 4. Discussion

Several reasons explain the differences between LTPAS practice regarding generation. The first reason is immigrants’ schooling. The young second and 1.5 generations have been educated in the Spanish education system, in which one of the compulsory subjects is physical education, a factor that is crucial for acquiring the habit of PAS practice. However, the young first generation have passed the limit of compulsory education, which means that many of them have been directly incorporated into the labor market without going through school. In our case, only 1.5% of first-generation immigrants are studying today. Moreover, as shown by Arjona et al. [48], barely 2% of foreigners who obtain a High School diploma will enroll at the University of Almeria, which means a high dropout in secondary school in favor of immediate incorporation into the labor market, drastically reducing leisure time. 

For that reason, the regression also highlights being a student and age as significant factors in explaining the variability of the index: the older the person, the less physically active, as immigrants are joining the labor market after leaving compulsory education and, therefore, either abandoning regular exercise practice, even in their leisure time, or undertaking it in an irregular and individual way.

Second, much of their socialization takes place in the educational system where values conducive to PAS are transmitted (which counteract those obtained through their families or origins), promoting a positive attitude towards LTPAS.

Third, 1.5- and second-generation immigrants have a social network, mainly created at school, which goes beyond their compatriots, allowing them to first see and then participate in LTPAS. However, first-generation youth have a lower social capital and rarely see other fellows to practice LTPAS, because they are working and exhausted after work.

African immigrants show lower participation in LTPAS. The explanation lies in the labor markets where many usually work: young Africans work mainly in intensive agriculture, with split shifts and weekend work; Latin Americans and Eastern Europeans are active in the services sector and construction, with working hours concentrated from 8 am to 15 pm, or 15 to 22 pm, with weekends off [50]. Furthermore, in the case of Africans, some cultural patterns such as religion should be taken into account. The Africans living in Andalusia derive from countries that practice the Islamic faith, where the role of physical activity, both in daily life and in the education system is secondary [38,46,48], a situation strongly reflected, as observed, in first-generation immigrants. Nonetheless, for the rest of the analyzed origins, the concept of body, the values of strength, achievement, and performance are very marked [15].

Stodolska and Livengood [41] show that the role of religion for Muslims is fundamental for defining leisure activities. The family acts as a transmitter of such values. During leisure time, sports activities or dances practices, therefore, are approved by religion. This research, however, recognizes that selective acculturation is not absolute, especially due to the role of generations and the education system.

Immigrant women [47,51], as well as Spanish women, presented lower levels of sports practice than men. As shown in other studies [25,43,52,53], cultural and economic factors are the main determinants for LTPAS participation. First, in addition to working (not at home) or studying, women bear the responsibility for housework and childcare and/or younger siblings. These duties prevent leisure time activities. 

Second, in the case of African Muslims there is pressure or lack of approval by the family or social group to dedicate free time to PAS. Control and attachment to traditional values are accentuated if we add the gender issue. Religious dictates are usually more demanding for women, especially in regard to their social life and activity outside home. For example, the LTPAS for Muslim women must be a secondary element and when practiced must fulfill many standards: clothing, type of exercise, etc. Furthermore, the practice of sport for this group, like others, has little social support [29], especially by the social norms that support the practice. All of this results in a lack of positive attitudes toward PAS [26,43].

Therefore, their exercise is confined either to taking their children/siblings for a walk in the parks or promenades in cities, or those activities that were practiced in their countries of origin or allowed by their cultural values. Therefore, it is easy to find examples of conflict between parents and teachers regarding the participation of girls in classes of physical education over the exercises performed or the clothing they must wear.

In addition to religion, women must overcome other constraints to develop PAS, especially those derived from family and professional roles. They are the ones who assume domestic responsibilities: childcare and household chores [52,53]. Consequently, their free time is much reduced for the practice of PAS, on top of the fatigue added by their double workday.

However, in recent years, immigrant women have been contributing significantly to the practice of physical exercise, primarily motivated by health benefits [29], beauty standards [10,11], and a range of activities more adapted to their schedules [54,55]. Monserrat [12] and the Spanish Ministry of Culture and Sports [56] show that the chief reason for physical exercise in Spain is to be in shape, followed by health, entertainment, and relaxation. Therefore, while there is a coincidence regarding health, it is not the case with the rest of these motivations. This reflects either less free time for leisure activities among immigrants or a different use of their free time.

Spaniards with a low social position practice less LTPAS [19,47]. A similar situation occurs among young immigrants settled in Andalusia. Bourdieu [57] and Bairner [58] show how people belonging to lower social classes make a different use of their free time from wealthier social groups. For example, instability and flexibility of jobs impede them from having a fixed schedule for any leisure activity, while a lack of resources impedes them from developing activities that require specific materials. Roberts [59] adds that even practicing the same activities, the meanings may be different. That is why Maza [60] recognizes that sport, in many cases, acts as a mechanism of social reproduction of the immigrant population. For example, Llopis [61] shows that for more than 60% of immigrants living in Spain who perform LTPAS, the activities consist of walking or team games, as compared to the Spanish (native) population, who perform a broader range of activities: swimming, fitness, running, skiing, and cycling. However, this fact leads, as Stodolska argues [30], to a demonstration effect, since immigrants wish to practice these types of activities because of their association with a high social status.

Among the contextual variables that explain variability is the identification rate of immigrants with their country of origin, also called endo-group favoritism, which consists of benefiting and valuing one’s own group more than another, according to attitudes, perceptions, or preferences, which determine, in some cases, discrimination or conflict [61,62].

Translated into LTPAS, immigrants are seen more as opponents than potential partners (as observed in sport, mass consumption, and entertainment), making the practice of mixed sports, and therefore intercultural communication through sports, difficult beyond schoolyards. Therefore, immigrants reduce their LTPAS practice to within their associations to compete with other immigrants from other associations or against the Spaniards in local leagues organized by themselves or local government. Today, the fact of organizing a sporting event involving people from multiple origins does not mean interculturality. This is achieved, among other things, by designing activities in which all individuals feel identified with the activity and not with the color of the shirt or flag. In other words, if PAS is developed solely by maintaining principles, rigid forms, and rules, without allowing, for example, different types or forms of practices within the same sport [62,63] in multicultural societies, as in Spain and Andalusia in particular, then sport will not achieve one of its main purposes: to integrate and facilitate communication. This is particularly the case in a country like Spain, where cultural and national diversity are recognized in the Constitution and where the different programs of integration, and their ideology, are committed to interculturalism. 

We finish this section by highlighting that in Spain only Dominguez [17] deals with the acculturation of immigrants through sport and has variables and goals in common with this study. However, in his research he does not use any means of measurement. Therefore, our proposed PASAI takes the challenge of becoming a valid device to study acculturation through sport. We also want to underline that the novelty of this measuring instrument, despite its reliability, can make its application elsewhere problematic. This is because the environmental characteristics, the structure of opportunities, the characteristics of the groups, the role of reception context in terms of leisure in general, and PAS in particular, are different in each country. Therefore, some of the variables that define the PASAI should be added or even eliminated, accordingly. 

## 5. Conclusions

First, Acculturation occurs in a segmented manner. 

Generation and origin are fundamental variables in explaining the process of acculturation [34]. According to our data, there is a positive association between generations 1.5 and 2 and LTPAS. This shows that, over time, the process of acculturation in LTPAS becomes more evident [12,20,23]. African immigrants are the group with the lowest participation in LTPAS.

Second, the index of acculturation (PASAI) is a positive aid for constructing a more accurate interpretation of social inclusion as it contributes to an overall understanding of migration. Besides being a new tool, it can become an instrument for comparison with other places and times. 

Third, since sport constitutes a means for intergroup relationships, the organization of sporting events should be encouraged beyond the school domain. In the same way, sport should enter the political agenda beyond the organization of competitive sporting events. Through the implementation of diversity policies according to the contexts in which they are applied, it could serve to improve coexistence.

## Data Availability

Not applicable.

## Appendix A

**Table A1:** Basic data of the research screening sample.

	Mean	Standard Deviation	Range
Age	17.6	2.9	15–20
Time in Spain	6.8	2.8	0–10
	N	%	
Gender			
Female	227	51.6	
Male	213	48.4	
Educational level			
Less than secondary	120	27.2	
Secondary or more	320	72.8	
Generation			
First	135	30.7	
Generation 1.5	191	43.5	
Second	114	25.8	
Origin			
África	126	28.7	
Europe no 15	64	14.5	
Europe 15	34	7.7	
America	190	43.2	
Asia	26	5.9	

**Table A2:** Dimensions and variables of the PASAI.

Dimensions	Item 1	Item 2
Mixed relations	Reason for PAS practice	Population group for regular PAS practice
Cultural references	Frequency of PAS practice	PAS type
Norms	Knowledge of PAS public offer	Participation in PAS extracurricular activities
Transnationality	Information about the country of origin	Sense of identity

Source: Own elaboration.

**Table A3:** Dimension frequencies of the PASAI.

			(%)
Mixed relations	Reason for PAS practice	- Health - Social relationships - Leisure - Competition - Adventure - Others	64.1 16.1 21.7 1 0.6 4.7
	Population group for regular PAS practice	- Alone - Most from my country - Most from Spain - Most from other countries - With all alike	42.3 31.6 7.2 4.9 14
Cultural references	Frequency of PAS practice	- Every day - 1–2 days a week - 3–4 days a week - 5–6 days a week - Irregular - Never	4.2 33.7 20.1 2.2 23.8 16
	PAS type	- From my country - From Spain - Universal	2.8 1.1 96.1
Norms	Knowledge of PAS public offer	- Yes - No	9.7 90.3
	Participation in PAS extracurricular activities	- Yes - No	14.4 85.6
Transnationality	Information about the country of origin	- Never - Less than once a month - Once a month - Several times a month - Once a week - Several times a week - Every day	13.2 16.6 22.9 28.4 15 22.1 10.2
	Sense of identity	- Only Spanish - As foreign and Spanish - Only foreign	0.7 63 36.3

Source: Own elaboration.

**Table A4:** PASAI according to origin (%).

PASAI	Sub-Saharan Africa	Eastern Europe	Latin America	Maghreb	Total
0	11.8	2.7	2	13.1	7.4
1	12.2	8.8	5.6	28.3	13.7
2	24.3	26.4	20.3	21.6	23.1
3	33.4	27.6	24.8	17.4	25.8
4	13.3	21.5	34.6	12.9	20.6
5	4.0	7.6	8.1	4.1	6
6	1.0	4.1	3.1	1.9	2.5
7	-	1.3	1.5	0.7	0.9
8	-	-	-	-	-
Total	100	100	100	100	100

Source: Own elaboration. X^2^ = 69.040; Gl = 21; *p* = 0.000.

**Table A5:** PASAI according to generation (%).

PASAI	First Generation	Generation 1.5	Second Generation
0	16.3	3.1	-
1	26.0	13.2	0.6
2	21.5	17.6	2.6
3	16.3	15.6	9.3
4	10.2	27.5	25.3
5	6.1	12.5	33.7
6	3.6	8.8	22.4
7	-	1.7	6.1
8	-	-	-
Total	100	100	100

Source: Own elaboration. X^2^ = 72.033; gl 21; *p* = 0.000.

**Table A6:** Effects of independent variables on the Acculturation Index (PASAI).

Variables		Model 1	Model 2
Individual Variables	Maghrebi origin	0.033 **	0.031 *
	Latino origin	0.212 **	0.209 **
	Eastern European origin	0.445 *	0.323 *
	Generation (First)	−0.584 *	−0.565 *
	Years of schooling	0.140 *	0.102 *
	Level of language skills (understanding and speaking)	0.236 *	0.221 *
	Incomes (less than €600 monthly)	−0.213 *	−0.197 *
	Subjective social class (high)	0.035 *	0.032 *
	House owner	0.061 *	0.061 *
	Muslims	−0.320 *	−0.280 *
	Christians	0.107 **	0.107 **
	Atheists	0.02 ***	0.022 ***
	Age	−0.534 **	−0.528 **
	Gender (male)	0.110 **	0.104 **
	Occupation (studying)	0.485 **	0.443 **
	Ideology (left-wing)	0.015 ***	0.015 ***
	Languages spoken	0.024 ***	0.023 ***
	Contact with origin (Yes)	−0.012 ***	−0.013 ***
	Idea of returning (affirmative)	−0.002 ***	−0.002 ***
Structural variables	Composition of the neighborhood (Spanish)	-	0.103 **
	Father’s educational level (illiterate)	-	−0.101 *
	Number of household members	-	−0.038 *
	Identified (foreigner)	-	−0.254 *
Coefficient R^2^		0.33	0.39

Source: Own elaboration. * *p* < 0.05; ** *p* < 0.01; *** *p* > 0.05.
